# Blended digital health intervention for adolescents at high risk with digital media use disorders: protocol for a randomised controlled trial within the Res@t-Consortium

**DOI:** 10.3389/fpsyt.2024.1478012

**Published:** 2025-01-20

**Authors:** Oliver Labrenz, Lucie Waedel, Michael Kölch, Susanne Lezius, Christina Wacker, Antonia Fröhlich, Kerstin Paschke, Rainer Thomasius, Olaf Reis

**Affiliations:** ^1^ Department of Child and Adolescent Psychiatry, Neurology, Psychosomatics, and Psychotherapy, University Medical Center Rostock, Rostock, Germany; ^2^ German Center for Child and Adolescent Health (DZKJ), Site Greifswald/Rostock, Rostock, Germany; ^3^ Institute of Medical Biometry and Epidemiology, University Medical Center Hamburg-Eppendorf, Hamburg, Germany; ^4^ German Center for Addiction Research in Childhood and Adolescence (DZSKJ), University Medical Center Hamburg-Eppendorf (UKE), Hamburg, Germany

**Keywords:** digital media use disorders, digital health intervention, youth at high risk, adolescents, child and youth welfare services

## Abstract

**Background:**

Digital media use disorder (DMUD) is a prevalent problem among young people, which can result in adverse consequences and functional impairments across multiple domains of life due to a persistent inability to regulate one’s use, which can lead to the development of psychological problems. In particular, children and adolescents who live in families that are part of the child and youth welfare system and receive support services are considered to be at high risk of developing mental disorders. It is less likely that these families will choose a therapeutic setting for the treatment of DMUD. The objective is to reduce DMUD-related symptoms and improve media use behaviour through the implementation of an app-based training programme.

**Methods:**

The efficacy of Res@t digital, initially conceived as an adjunct to child and adolescent psychiatric treatment, is to be evaluated for n= 32 children and adolescents with a media use disorder or at risk of developing this disorder, and their families enrolled in child and youth welfare services. The efficacy of the app will be evaluated in a randomised controlled trial with a waitlist control group. The primary outcome is the reduction of DMUD symptoms over a 20-week period following the onset of app training. Secondary outcomes include EEG measurements and changes in standardised psychopathological variables.

**Discussion:**

Should the Res@t app prove efficacious when compared to a waitlist control group, it would constitute an evidence-based intervention for the treatment of DMUD in children and adolescents. For high-risk families, the app could serve as a motivational tool to prompt action regarding potential DMUD and facilitates access to therapeutic facilities.

**Clinical trial registration:**

https://drks.de, identifier DRKS00033379.

## 1 Introduction

### 1.1 Background and rationale

As time progresses, the public healthcare system is confronted with ever new phenomena of cultural and technological progress. Such advances have been seen in the recent past in the digital media sector, where availability and attractiveness of digital media use are increasing worldwide, apparently not without harbouring health risks. So-called “Digital media use disorders” (DMUDs) refer to behavioural addictions in which the use of digital media leads to a dependency and persistent impairment of psychosocial functioning over a certain period of time (1–3). This is an umbrella term that can be used to summarise different types of media (e.g. computer, smartphone, television), usage patterns (e.g. playing video games or gaming, watching video streams, social networking) and connectivity (online on the internet or offline). Global prevalence rates (with considerable regional differences) from an international meta-analysis by Meng et al. (4) show that 17.4% of the population have a social media addiction and 6.0% a game addiction. Furthermore, 27.0% have a smartphone addiction and 14.2% have an internet addiction. As a result, the first specifically described DMUD, “Gaming Disorder” has been included in the 11th version of the International Classification of Diseases (ICD-11; icd.who.int/browse/2024-01/mms/en) with the code 6C51 and the extension predominantly online (.0) or offline (.1). 6C51 is met with all of the following criteria related to gaming behaviour: impaired control over temporal or situational aspects, neglect of other interests or activities in favour of the behaviour, persistent behaviour despite evidence of harmful consequences, manifested over a considerable period of time (continuously or in recurring episodes for e. g. 12 months), and significant impairment in areas of psychosocial functioning (e.g. family, friends or education). Other DMUDs such as social media use disorder and streaming disorder are listed under the code 6C5Y with the generic term “Other specified disorders due to addictive behaviours”. If the criteria for a DMUD are not met, but risky user behaviour is present, this can be coded with QE22 for “Hazardous Gaming” or QE2Y “Problems with other specified health-related behaviours”.

With maturing brains, adolescents are a particularly vulnerable group for DMUD due to their still developing cognitive control and responsiveness to reinforcing systems, which are widely used in digital media today (5). Especially impulsivity and depressive rumination facilitate Internet addiction in adolescents (6). The younger generation growing up with information and communication technology is also frequently exposed to digital media, which leads to multidimensional interactions, particularly with regard to the causes of and attempts to cope with psychiatric disorders (7–11). Digital media presents a number of advantages: lower barriers when it comes to difficulties in social communication and interaction, immersion allows the “escape” from stressful events into an alternative reality and compensates for the lack of reinforcement in everyday life (12, 13). In particularly severe cases, DMUD gives rise to the phenomenon of “hikikomori”, initially documented in Japan. Hikikomori individuals isolate themselves in their homes, only venturing out on rare occasions. This represents a severe form of social withdrawal in which digital media play a significant role (14).

Social factors, including poverty, social exclusion, a lack of parental competence and supervision, and inconsistent parental behaviour, have been identified as playing a role in the development of Internet Gaming Disorder (IGD) (15). In particular, in school-aged children, family factors such as family violence and poor parental care have been identified as major risk factors for IGD (16). It is often the case that children growing up in environments more susceptible to the aforementioned risk factors are referred to child and youth services for care. The provision of child and youth welfare services plays a crucial role in the well-being of children. Children placed in child welfare systems are often characterised by a high prevalence of behavioural problems that are often associated with multiple family problems, such as parental mental health issues or substance use disorders (17, 18). It is recommended that young people be provided with more accessible psychological support services that offer measures to promote healthy coping mechanisms (19). In light of the recent coronavirus pandemic, the development of appropriate programmes to prevent and reduce behavioural addictions such as DMUDs is of particular importance (20–22).

In order to meet the specific needs of adolescents with emotional disabilities and youth at special risk, the incorporation of multimedia elements and electronic performance support systems in prevention and intervention for these subgroups has proven to be more effective than traditional interventions (23, 24). In particular, for the treatment of depression and anxiety, internet-guided and unguided digital E-mental health interventions have been demonstrated to be an effective form of treatment for adolescents, with the potential to reduce symptoms and promote well-being (25–27). A blended approach, which combines online intervention with guided contact during digital training, offers multiple advantages. This is particularly relevant from the perspective of young people and their families in the context of gaming disorder (28, 29). As part of the Res@t-Consortium (www.uke.de/projekte/resat), an app was developed that is tailored to the specific needs of young people with DMUD, serving as a digital counterpart to the CBT-based Res@t offline therapy program. The goal of this study is to demonstrate feasibility and effectiveness of the intervention for adolescents at high risk with DMUD or hazardous use pattern, who are to be reached in a blended approach and motivated to participate and train with the app. Adolescents at high risk was defined as 1) family was approached by the youth welfare service because of family-related problems (e.g. long-standing conflicts, family violence, parents or youth seeking help, school absenteeism), or 2) the family had received a recommendation for contacting the youth welfare service (from school, child and adolescent psychiatry, police), or 3) the adolescent had special needs for schooling (e.g. attention problems, hyperactivity or learning difficulties). The theoretical embedding and assessment of the effectiveness of the treatment is based on Prochaska’s Transtheoretical Model of Change in Behaviour (29, 30), which is one of the most frequently used models in this field. It describes an experience of different phases of change, which begins with the stage of Precontemplation (lack of intention to change behaviour) and leads through Contemplation (intention to change behaviour in the future) to the stage of Preparation (first steps towards behaviour change), Action (performing the behavioural change) and Maintenance (maintaining the behaviour) stages. The respective individual stage is an important factor to consider when evaluating the impact of interventions.

Moreover, for a more comprehensive understanding of DMUD, its neurobiological underpinnings of these behavioural addictions should be investigated, in addition to measures of experience and behaviour. This kind of multidimensional approach is not only in line with the Research Domain Criteria (RDoC) approach, which considers psychiatric disorders through various constructs on several units of analysis (31), but also offers a chance to detect neurobiological predictors of effective treatment. In order to follow the transdiagnostic approach of the RDoC, not only DMUD diagnoses, but also hazardous use patterns that do not yet justify a DMUD diagnosis, as well as a wide range of comorbidities are included in this study. Especially in the view of DMUD as dysfunctional coping with e.g. emotional stress, “pure” DMUD diagnoses (no comorbidities or precursors of the diagnosis) would exclude parts of the target group in need of support and distort the therapeutic effect of the intervention in real practice. For this reason, no comorbidities are excluded, with the exception of those that make participation in the study impossible. The domains investigated are addiction-related, such as positive valence (reward responsiveness, learning, and valuation) and cognition (cognitive control). In order to take the neurobiological basis into account and investigate fundamental mechanisms, the analysis levels circuits and physiology are examined by means of electroencephalography (EEG) in addition to self-reports, behaviours and paradigms.

### 1.2 Objectives and trial design

In accordance with the Res@t consortium’s plan, the efficacy and effectiveness of the digital health intervention “Resource-Strengthening Training for Adolescents with Problematic Digital-Media Use and their Parents” (Res@t digital) will be examined. The aim of this training is to reduce the mental health problems associated with DMUD in adolescents and to strengthen parental self-efficacy. In order to shed light on various aspects of such a novel care programme, high-risk groups within child and youth welfare services are being investigated in this additional study alongside a main study (31). As an addition, this study supplements the main study by recording potential neurophysiological changes using EEG.

Participants will be randomised-controlled with a 1:1 allocation ratio into two arms of parallel groups, consisting of an intervention group (IG) and a waitlist control group (CG). Both groups receive the treatment as usual (TAU), meaning the standard child and youth welfare programme, while the IG additionally receives the Res@t digital intervention during the study period. Each subject in the CG will be given the opportunity to receive the training after full participation following the last data collection.

Our trial aims to test the hypothesis of the superiority of Res@t digital combined with child and youth welfare service through a greater reduction in the symptoms of the most prominent DMUD or hazardous use pattern in the individual adolescent, compared to child and youth welfare service alone. Primary hypothesis: Res@t+TAU reduces symptoms of specific DMUD in adolescents compared with TAU alone, measured as a group-by-time interaction over 5 measurement points from screening to a 10-week follow-up. Secondary hypotheses: a) Res@t+TAU reduces symptoms of specific DMUD in adolescents as assessed by their parents compared with TAU alone, measured as a group-by-time interaction over 5 measurement points from screening to a 10-week follow-up. b) Res@t+TAU reduces symptoms of specific DMUD in adolescents assessed by authorised personnel compared with TAU alone, measured as change from screening to post-intervention. c) Res@t+TAU will have a beneficial influence on several observation-based constructs related to DMUD in adolescents (improved readiness to change and sleep quality), in parents (improved life satisfaction and family self-efficacy) and in both adolescents and parents (reduced stress levels and improved family functioning and mindfulness) compared with TAU alone, measured as change from baseline to post-intervention. d) Res@t+TAU reduces DMUD typical or potential markers in the EEG compared with TAU alone, measured as change from baseline to post-intervention.

## 2 Methods

### 2.1 Study setting

The study presented here is one of two additional studies accompanying the main Res@t study (32). It is carried out by the University Medical Centre Rostock and will be conducted in the urban area of Rostock and the district of Rostock (approx. 200 and 220 thousand inhabitants) in the German state of Mecklenburg-Western Pomerania. We are planning recruitment for the period from May 2024 (first participant in) to March 2025 (last participant in). The study presented here takes an approach, where child and youth welfare providers identify adolescents who are at particularly high risk for DMUD. We expect this group to be low on motivation, calling for a more blended approach wherein extended face to face contacts are necessary to maintain compliance.

### 2.2 Eligibility criteria

Inclusion criteria for participants:

- Recipients of or the recommendation to receive child or youth welfare or have special needs for schooling.
- 10 to 19 years of age (WHO definition of adolescence).
- Cut-off for disordered or hazardous media use in the Gaming Disorder Scale for Adolescents/for Parents (GADIS-A/-P), Social Media Disorder Scale for Adolescents/for Parents (SOMEDIS-A/-P) and Streaming Disorder Scale for Adolescents/for Parents (STREDIS-A/-P) is reached (see primary outcome).
- Fulfilled criteria for disordered or hazardous media use according to ICD-11 criteria (6C51, 6C5Y, QE22, QE2Y).
- Written informed consent is given (for adolescents under the age of 16, the informed consent of the legal guardian is also necessary).

Or

- Are a parent/legal guardian of a participant fulfilling the criteria above.
Exclusion criteria for participants:
- Acute severe psychiatric disorders with a symptom burden that prevents participation in the study (i. e. psychotic disorders or disorders due to substance use).
- Pervasive developmental disorders (i. e. autism spectrum disorder).
- Acute suicidality.
- Inability to understand the study instructions (i. e. severe disorders of speech or language, diminished intelligence or lack of german language skills).

### 2.3 Recruitment

Access to the sample is mainly via the employees of child and youth welfare services and facilities. They establish contact between potential participants and our study team. As soon as contact has been established, the study team takes on all tasks relevant to the study and the employees of the child and youth welfare services have no further obligations. Furthermore, recruitment takes place in the district and meeting centres of the city of Rostock, as these are places that are frequented by adolescents from problematic backgrounds on the one hand and are accompanied by child and youth welfare staff in these facilities on the other. Finally, the work groups on child and adolescent psychiatry and addiction disorders in the city and district of Rostock are also included by the corresponding psychiatry coordinators and potential participants who are in child and youth welfare services are recruited. Once the study team has received the contact details of willing participants from the youth and social services, the participants and legal guardians provide informed consent to the study team. The study team then administers questionnaires at all-time points (screening, baseline, interim, post-intervention, follow-up 1 and 2) and conducts the clinical interviews (baseline and post-intervention).

### 2.4 Intervention

The IG receives the app-based resource-strengthening adolescent and parent training programme (Res@t digital) after completing baseline assessment. The training consists of 10 modules: a first week training start following two weeks of psychoeducation, five weeks of specific contents, a one week relapse prevention and finally a booster session. A new module is activated every week, whereby the booster module is only activated 5 weeks after module 9 in week 15 of the training. The specific contents differ for adolescents (Res@t-A) and parents (Res@t-P), with the exception of the module on communication. Adolescents receive modules with specific contents on health and sleep hygiene, self-care, dealing with emotions and social relationships, while parents receive modules on developmental tasks and parenting styles, implementing rules, applying rules and family health. Depending on the type of dominant DMUD or hazardous use pattern, the content of the app is adapted to it. In addition, participants can use a diary in which they can enter daily times of media use, mood, activities, daily structure and sleeping times. For a detailed description of the training and the app contents, see Paschke et al. (32). Participation by parents is encouraged but not mandatory. Participants in the CG are assessed in the same way as in the IG using questionnaires and EEG (see outcomes below), but receive Res@t-A/P only after completing the last assessment and on an optional basis.

### 2.5 Outcomes

#### 2.5.1 Primary outcome

The primary outcome is the difference in the severity of specific DMUD or hazardous use pattern between IG and CG within 20 weeks of enrolment, measured at 5 time points (screening, interim, post-intervention, follow-up 1 and 2) at 5-week intervals. It is assumed that the group using the app (IG) shows bigger decreases in DMUD or hazardous use pattern compared to the group without (CG). In the event that an individual shows more than one DMUD or hazardous use pattern, the severity is operationalised by the supervising study team with the most severe type of DMUD or hazardous use pattern (gaming, social media or streaming). In order to assess gaming, social media and streaming as key areas of digital media consumption, the GADIS-A/-P, SOMEDIS-A/-P and STREDIS-A/-P are used to identify disordered or hazardous media use (33–38). All three questionnaires are based on the ICD-11 criteria for gaming disorder and other specified disorders due to addictive behaviours, which are specified here as social media use disorder and streaming disorder. The questionnaires consist of 4 items cognitive-behavioural symptoms (CBS) of problematic media use, 5 items negative consequences (NC) and one item on the frequency of these difficulties, with the exception of STREDIS-A/-P, in which CBS and NC account for 3 and 6 items respectively. If the cut-offs for CBS and NC are reached and the time criterion is met, disordered media use is indicated. If only the cut-off for CBS is reached, but not for NC, hazardous media use is assumed. If only NC but no CBS is present, another mental disorder may be present. In addition, a clinical interview to diagnose the presence of disordered or hazardous media use according to ICD-11 is assessed (at Screening and Post-Intervention) by authorised personnel. In order to take the high-risk sample into account, the assessment times were set more closely compared to the main study (32) and the baseline, post-intervention and follow-up assessments were supplemented by a measurement with GADIS-A/P, SOMEDIS-A/P and STREDIS-A/P interim (5 weeks after baseline in the middle of training) and an additional follow-up (5 weeks after the end of training and 5 weeks before the original follow-up). The interval of the DMUD questionnaires is therefore shortened from every 10 weeks to every 5 weeks (see participant timeline).

#### 2.5.2 Secondary outcomes

As for secondary outcomes we assume that the use of the app will have a beneficial influence on several observation-based constructs related to DMUD. For psychopathological symptoms of the adolescents and perceived stress by adolescents and parents we assume a bigger decrease for the IG. The Strengths and Difficulties Questionnaire (SDQ) measures the psychopathological symptom burden of adolescents using five items on each of five subscales: emotional symptoms, conduct problems, hyperactivity/inattention, peer relationships problems and prosocial behaviour (39–41). The self-assessment exists from 11 years of age and a parallel external assessment by parents from 4 years of age. A slightly age-adapted version is available for 18 year olds and older. Adolescents are asked about the last six months in the screening and about the last month in the post-intervention. Parents only complete the SDQ-f at screening and were also asked about the last six months. The perceived stress of adolescents and parents within the past month is assessed using the 10 items of the Perceived Stress Scale (PSS-10) (42, 43). Analogous to the Transactional Theory of Stress and Coping, the scales Perceived Helplessness (primary appraisal; assessment of the situation and its stressors) and Perceived Self-Efficacy (secondary appraisal; assessment of resources and coping strategies) are formed (44). The phrasing was slightly adapted for adolescents in this study. The PSS-10 is measured at pre- and post-intervention.

On the other hand, we expect a greater increase in the IG for family functioning, family communication and mindfulness in adolescents and parents as well as an increase in family self-efficacy and quality of life of the parents. Family functioning is assessed using the five items giving the questionnaire its name: Adaptability, Partnership, Growth, Affection and Resolve, referred to as Family APGAR (45, 46). The family communication scale (FCS) measures “the act of sharing ideas, participating in decision making, and expressing feelings among members as a family unit” through ten items by self-assessment (47–49). Family functioning and communication are self-assessed by adolescents and parents. Family self-efficacy in parenting is measured by parents in the questionnaire with the same name (Familiäre Selbstwirksamkeit [FSW]) using nine items (50). The Mindful Attention Awareness Scale (MAAS-5) measures mindfulness (a state of mind characterised by receptivity, in which the subject is able to observe their thoughts, feelings and surroundings non-judgementally, thereby being present in the moment) in five items in adolescents and parents (51, 52). The Ulm Quality of Life Inventory for Parents (ULQIE) is used to assess parents’ life satisfaction over the last seven days (53). The 29 items of the ULQIE are partially incorporated into the subscales of physical and daily functioning, satisfaction with family support, emotional strain due to the child’s illness, self-development and well-being. All of the above mentioned questionnaires are collected at baseline and post-intervention.

With regard to the stages of change, we expect that adolescents in both groups will initially be in the stages of precontemplation or contemplation. After the intervention, more adolescents in the intervention group should be in the action stage than in the control group. Moreover, the influence of adolescent motivation should be explored as these variables should be modelled as covariates of change in DMUD or hazardous use pattern. Based on Prochaska’s Transtheoretical Model of Change in Behaviour (29, 30), we identify the stage of behaviour change in adolescents by means of the questionnaire for the assessment of readiness to change (Fragebogen zur Erfassung der Veränderungsbereitschaf [FEVER]) (54). The scales Precontemplation, Contemplation, and Action, each with eight items, are collected through self-assessment at baseline and post-intervention by the adolescents. The Preparation and Maintenance stages are not included in the questionnaire, as these are practically less informative.

Further, we expect an increase in sleep quality and a decrease in sleepiness and severity of insomnia among adolescents. Adolescents self-assess their sleep quality through the nineteen items of the Pittsburgh Sleep Quality Index (PSQI), their sleepiness in eight items of the Epworth Sleepiness Scale for Children and Adolescents (ESS-CHAD), and their severity of insomnia in seven items of the Insomnia Severity Index (ISI) (55–59). The sleep quality assessed by the PSQI can be determined from a combination of 7 components: subjective quality, latency (time required to fall asleep), duration of sleep, efficiency (ratio between time in bed and actual sleep), disturbance, use of sleep medication, and daytime dysfunction (e.g. due to fatigue or low activity level). The PSQI and ESS-CHAD reflect assessments over the last four weeks, while the ISI reflects assessments within the last two weeks.

**Figure 1:**
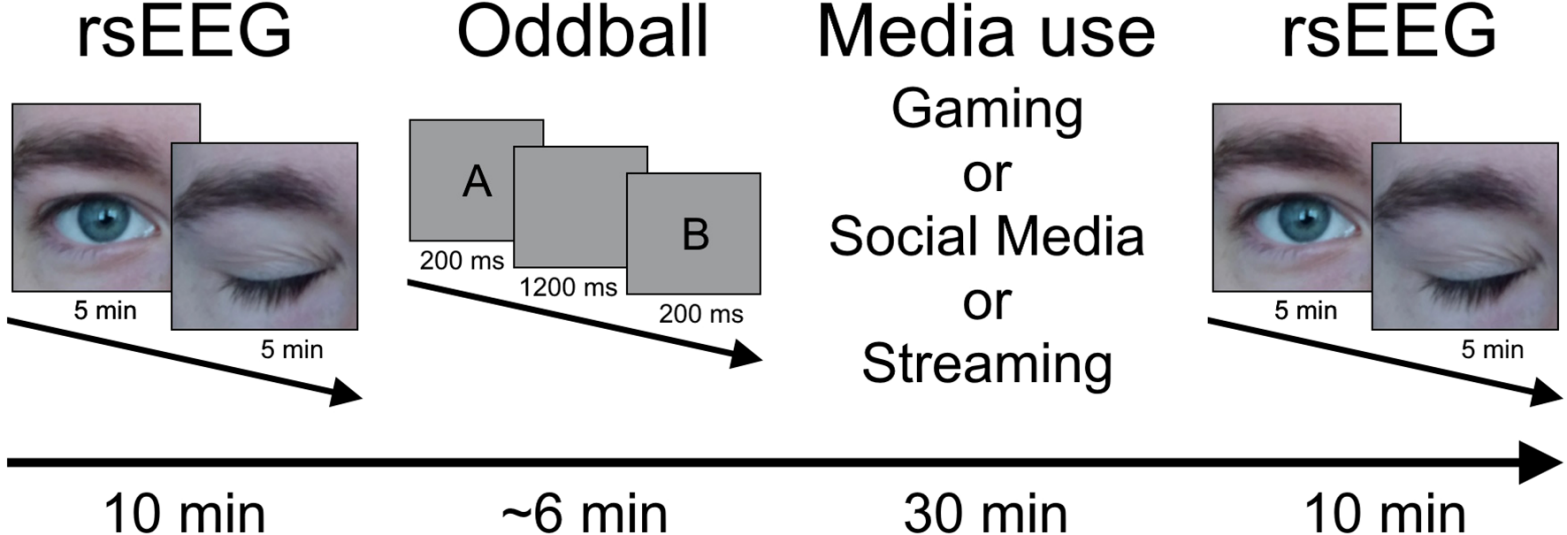
Procedure for the EEG assessment. rsEEG, resting-state EEG.

To measure neurophysiological characteristics of brain activity, an EEG is used. For this purpose, a resting-state EEG (rsEEG) with 5 minutes of eyes open and 5 minutes of eyes closed is recorded at the beginning, followed by approx. 6 minutes of an oddball paradigm. A 30-minute sequence of a favoured media related activity (e.g. gaming) follows. The EEG is therefore carried out in the participants’ homes using a mobile EEG. The EEG ends with another rsEEG identical to the one at the beginning of the recording (see **Figure 1**). 45 minutes are planned for the preparation of a 32-channel montage. The oddball task is active visual and administered as described in Kappenman et al. (60). Participants are presented with the letters A, B, C, D and E in random order in a trial. In each block, one of these letters is defined as a target, which must be distinguished from the nontargets by pressing the up and down arrow keys using the dominant hand. There are a total of 5 blocks, each with 40 trials, in which each letter is presented 8 times. Each stimulus is presented for 200 ms and the inter-stimulus interval is 1,200 to 1,400 ms. The first EEG is carried out in the period from screening to the start of training and a further EEG after the end of training.

There is already a modest number of EEG studies that investigate neurophysiological measures of DMUD in the EEG and qualify as potential biomarkers of DMUD and its therapy response (61, 62). As these studies showed, people with IGD exhibit reduced power in the beta frequency band and increased power in the lower frequency bands of delta and theta in the rsEEG. In addition, a hyperconnectivity of the default mode network (DMN) and reward/salience network (RSN) appeared in the rsEEG of IGD subjects (63). In the domain of ERPs, the components of N2 and P3 showed increased negativity and positivity as markers of IGD (64, 65). Therefore we assume several EEG parameters to be associated to the intervention:

- Decreasing theta/beta-ratio in Power Spectral Density of

the rsEEG.

- Decreasing connectivity within the DMN and RSN.

- Decreasing negativity N2 in the oddball task.

- Decreasing positivity P3 in the oddball task.

#### 2.5.3 Additional variables

Additional variables collected concern socio-demographic information, media rules, adolescent and parental media use, parental symptom burden and parenting style (66). Parental symptom burden is assessed at baseline by self-assessment of the nine items in the Patient Health Questionnaire (PHQ-9) for depressiveness and the seven items in the Generalizied Anxiety Disorder Scale (GAD-7) for anxiety within the last two weeks (67–70). Parents assess their parenting style in the Parenting Style Inventory (Eltern-Erziehungsstil-Inventar [EEI]) on the ten-item scales love, discipline, autonomy and on the seven-item additional scales cooperation with partners and cooperation with school, teachers and carers also at baseline (71). The religiosity scale is not surveyed. In addition, data on app usage behaviour is collected in regard to the number of app usage sessions, days, weeks, quests started, quests completed, mindfulness exercises observed, calendar entries, the relative completion of modules and the complete training.

### 2.6 Sample size

Calculating the sample size for the primary outcome of the change in the severity of DMUD or hazardous use pattern between the intervention and control group over 5 measurement points is based on a mixed ANOVA with repeated measures and a within-between interaction. Assuming an effect size of 0.20, alpha error probability.05 and power.8, GPower 3.1.9.7 calculated a total sample size of 32 subjects with 16 per group. Due to the high-risk conditions of the target group, we assume a drop-out rate of 50%, which results in a total of 64 subjects to be recruited.

### 2.7 Incentives

We expect the adolescents in our target sample of child and youth welfare programme to be less motivated than the participants from the clinical setting of the main study and are therefore pursuing a stronger and more consistent incentive strategy. Potential adolescent study participants already receive a €5 voucher for the screening, regardless of whether they will take part in the study or not. Adolescents receive €10 each for the complete baseline and post-intervention questionnaires and €5 each for the shorter GADIS/SOMEDIS/STREDIS-A questionnaires at interim, follow-up 1 and 2. The closer timing of the assessments results in a higher reward frequency.

### 2.8 Assignment of interventions and blinding

Allocation to IG or CG is carried out by our consortium partner at the University Hospital Schleswig-Holstein Kiel, whose participants are randomised by ourselves in return. A computer-generated central randomisation list with variable block lengths, will be created by a project-independent employee of the Institute of Medical Biometry and Epidemiology at the University Medical Center Hamburg-Eppendorf. Since the CG does not receive any sham treatment and the randomisation as well as the installation and implementation of the app is coordinated by the study team, there is no blinding.

### 2.9 Data collection, management, and analysis

The data is collected continuously using the Res@t app with the ISO-certified Embloom platform as the backend. Furthermore, the PsychoEQ programme, which facilitates the collection of questionnaire data via mobile phones, tablets and personal computers, is employed. All data pertaining to participants will be pseudonymized.

**Figure 2:**
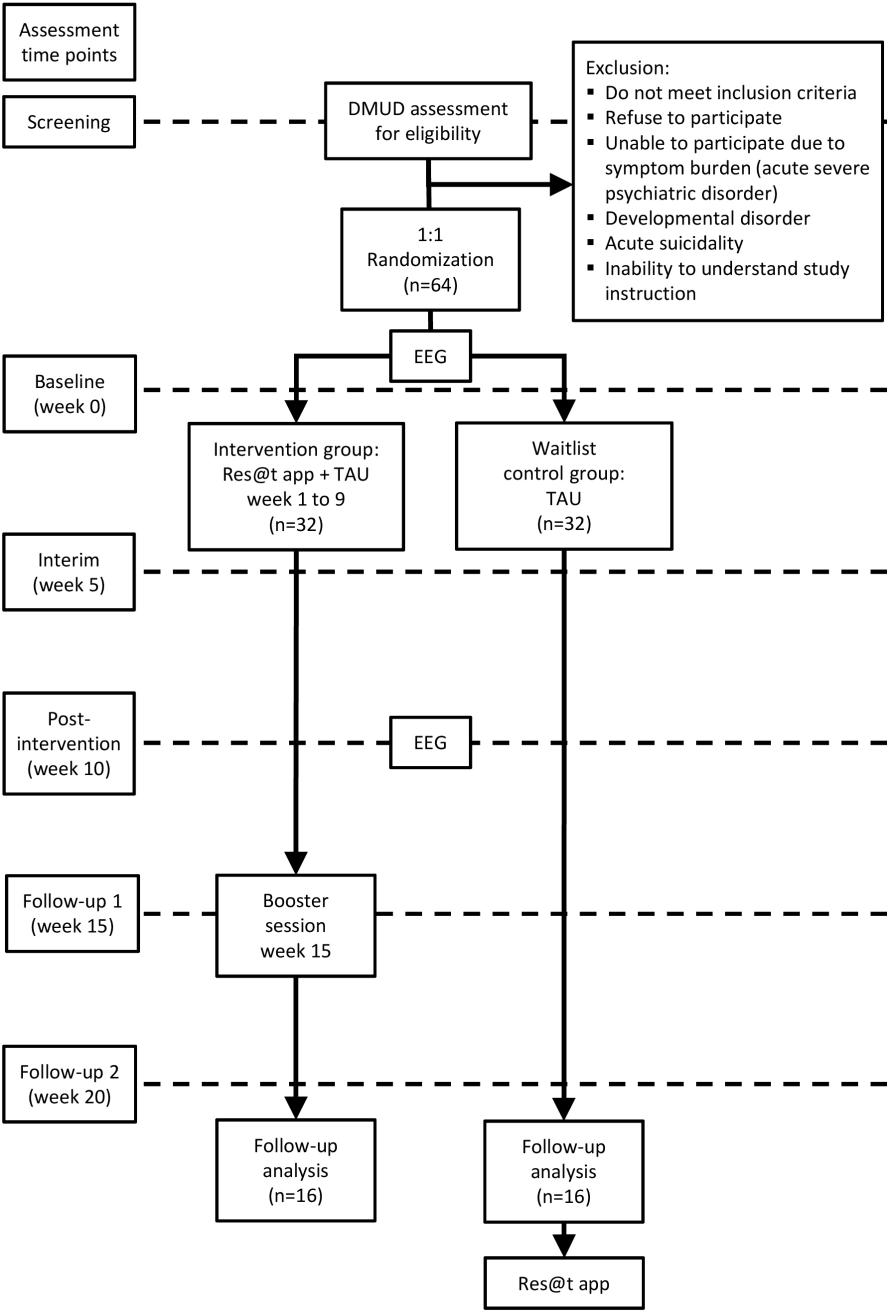
Study flowchart of participants. DMUD, digital media use disorder; EEG, Electroencephalography; TAU, treatment as usual.

Descriptive statistics are presented separately for each group and for the total sample. The data will be analysed using IBM SPSS 28 Statistics. A complete-case analysis will be conducted on the main outcome variable, the specific DMUD score, which will be evaluated at five points in time: baseline, interim, post-intervention, follow-up 1, and follow-up 2 (see **Table 1** and **Figure 2**). In order to determine whether there are notable differences in the impact of the treatment, a mixed ANOVA will be used, with the specific DMUD scores serving as the dependent variable and time and group serving as the within-subject factors and between-subject factors, respectively.

**Table 1:** Measurement time points.

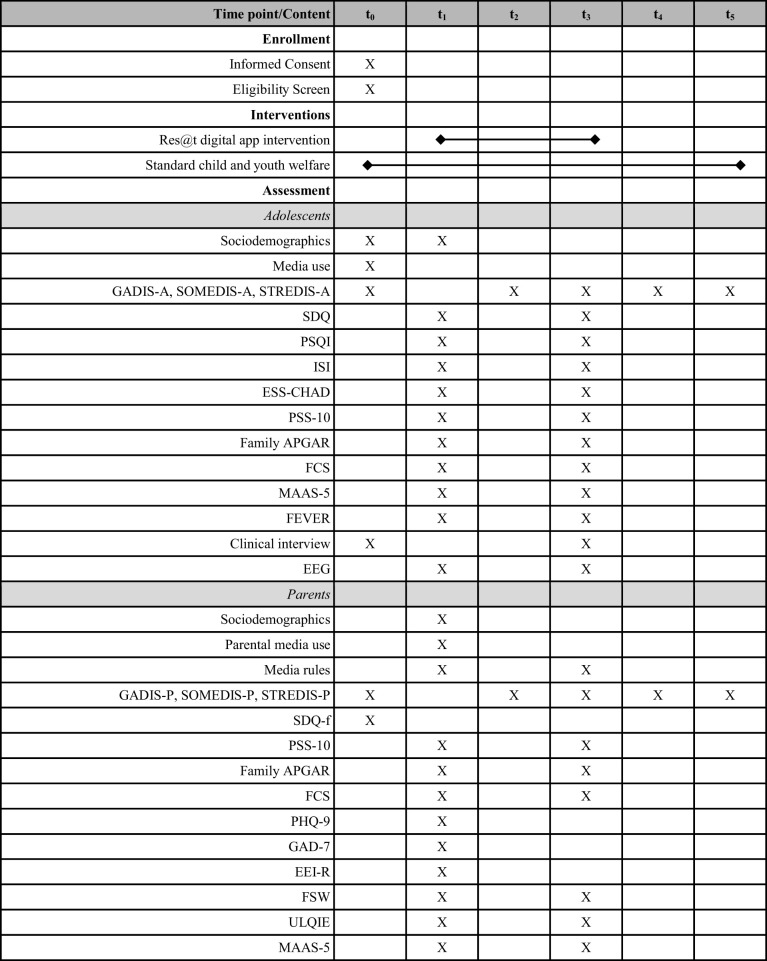

Time points: t_0_ = Screening (< week 0), t_1_ = Baseline (week 0), t_2_ = Interim (week 5), t_3_ = Post-intervention (week 10), t_4_ = Follow-up 1 (week 15), t_5_ = Follow-up 2 (week 20). GADIS-A, Gaming Disorder Scale for Adolescents; SOMEDIS-A, Social Media Disorder Scale for Adolescents; STREDIS-A, Streaming Disorder Scale for Adolescents; SDQ, Strength and Difficulties Questionnaire; PSQI, Pittsburgh Sleep Quality Index; ISI, Insomnia Severity Index; ESS-CHAD, Epworth Sleepiness Scale - Children and Adolescents; PSS-10, Perceived Stress Scale; Family APRGAR, Family Functionality; FCS, Family Communcation Scale; MAAS-5, Mindfulness Attention Awareness Scale; FEVER, Questionnaire for the Assessment of Readiness to Change; EEG, Electroencephalography; GADIS-P, Gaming Disorder Scale for Parents; SOMEDIS-P, Social Media Disorder Scale for Parents; STREDIS-P, Streaming Disorder Scale for Parents; SDQ-f, Strength and Difficulties Questionnaire – External Assessment; PHQ-9, Patient Health Questionnaire; GAD-7, Generalized Anxiety Disorder Questionnaire; EEI-R, Parenting Inventory - Revised; FSW, Parental Self-Efficacy; ULQIE, Ulm Quality of Life Inventory for Parents of Chronically Ill Children.

In the event of missing values, a sensitivity analysis will be conducted in accordance with intention-to-treat principles. Missing values will be assumed to be missing at random and handled using the multiple imputation method. A baseline-adjusted linear mixed model will be calculated with random intercept for patient, group and timepoint as well as their interaction as main effects and respective baseline value as covariate. The threshold for statistical significance of the primary outcome is set at p < 0.05. Further outcomes are analysed in an exploratory manner.

## 3 Discussion

The Res@t digital intervention aims to close a gap in the treatment and care of adolescents with DMUD and their parents. The aim of this study is to test the effectiveness and efficacy of an evidence-based training programme for high-risk groups such as adolescents in child and youth services. In this kind of setting, the DMUD or hazardous use pattern is embedded in highly stressed adolescents and family members, whereby awareness of the problem and motivation for treatment as well as family support regarding the DMUD are likely to differ from adolescents who are undergoing primarily treatment. It is possible that the highly individual circumstances and conditions in which children and young people supported by youth and social services live may limit the generalisability of the findings on the usability and efficacy of Res@t digital. The same applies to the subsample concerning school-related difficulties. Nevertheless, the efficacy of Res@t digital will be further examined in the main study (32) of outpatients diagnosed with DMUD, recruited from psychotherapeutic and psychiatric practices and hospitals. To address the presumably harder-to-reach high-risk target group, we use a high dose of reinforcement in the form of gift cards every five weeks. It is hypothesised that this reinforcement system will be effective when used in conjunction with a blended approach, in which personal contact is intended to ensure the successful implementation of the intervention. Study staff will then support adolescents and parents with motivational barriers, technical issues, or organising app implementation. Combining digital and face-to-face components to address the various challenges of participation may be innovative, but not universally effective. For example, adolescents with high levels of stress or low motivation may benefit less from the digital component, or face-to-face contact may be a barrier for those who are socially averse. Furthermore, the lack of blinding in a waitlist design could lead to participants in the IG being influenced by expectations of their group allocation, leading to placebo effects and biased results. Participants in the CG could feel disadvantaged, which could change their perception and response to the use of digital media. In addition, the lack of intervention could lead to reduced adherence if participants feel inappropriately treated. Another limitation is the acquisition of neurophysiological parameters using EEG in the participants’ homes rather than under laboratory conditions. This results in a higher validity of the media use measures, but may lead to an overall lower reliability caused by environmental factors. Despite the limitations mentioned, a positively evaluated Res@t app can represent a low-threshold treatment option compared to outpatient and inpatient treatment and serve as a motivational tool to facilitate entry into further treatment in a therapeutic setting. The evaluation will also show whether the combination of Res@t digital with the blended approach and reinforcement strategies has the potential to be an effective treatment option for hard-to-reach adolescents at high-risk, underscoring the need for real people to accompany the digital.
